# Supplementary material for: Synthesis and Characterization of Amphiphilic Diblock Polyphosphoesters Containing Lactic Acid Units for Potential Drug Delivery Applications
Source: Molecules. 2023 Jul 6;28(13):5243. doi: 10.3390/molecules28135243 (PMC10343241; doi:10.3390/molecules28135243)
Supplement: Supplementary file 1 [file molecules-28-05243-s001.zip › molecules-2457905-supplementary.pdf]

Synthesis and Characterization of Amphiphilic Diblock Polyphosphoesters Containing Lactic Acid Units for Potential Drug Delivery Applications

Tatsuya Sakuma[1], Kimiko Makino[1], Hiroshi Terada[1], Issei Takeuchi[1,2*], Violeta Mitova[3], Kolio Troev[1,3*]

[1]Faculty of Pharmaceutical Sciences, Tokyo University of Science, 2641, Yamazaki, Noda, Chiba 278-8510, Japan

[2]Faculty of Pharmaceutical Science, Josai International University, 1 Gumyo, Togane, Chiba 283-8555, Japan

[3]Institute of Polymers, Bulgarian Academy of Sciences, Sofia 113, Bulgaria

Supplementary Data:

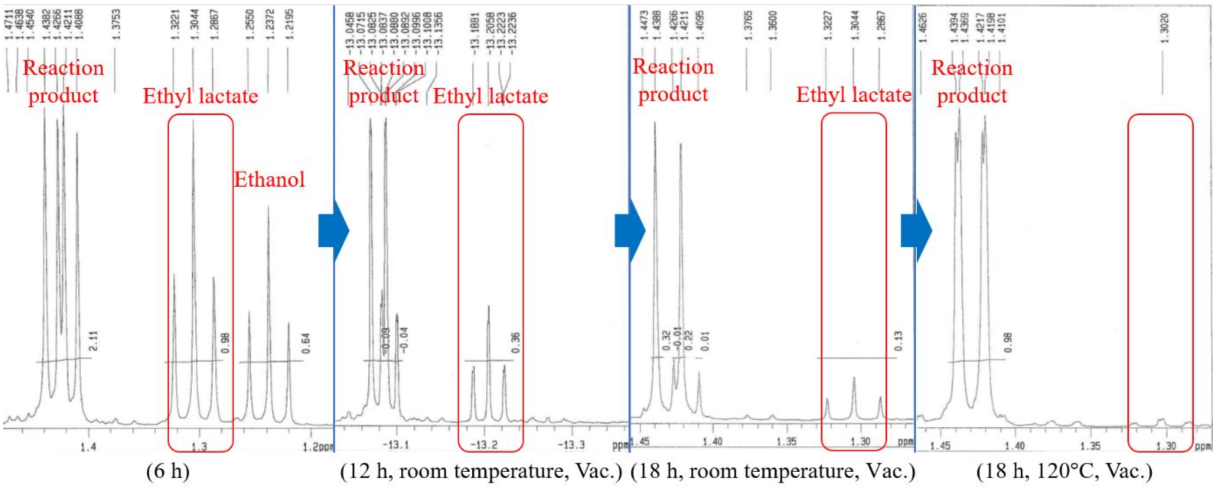

Figure S1. Reaction progress based on ^{1}H NMR spectroscopy.

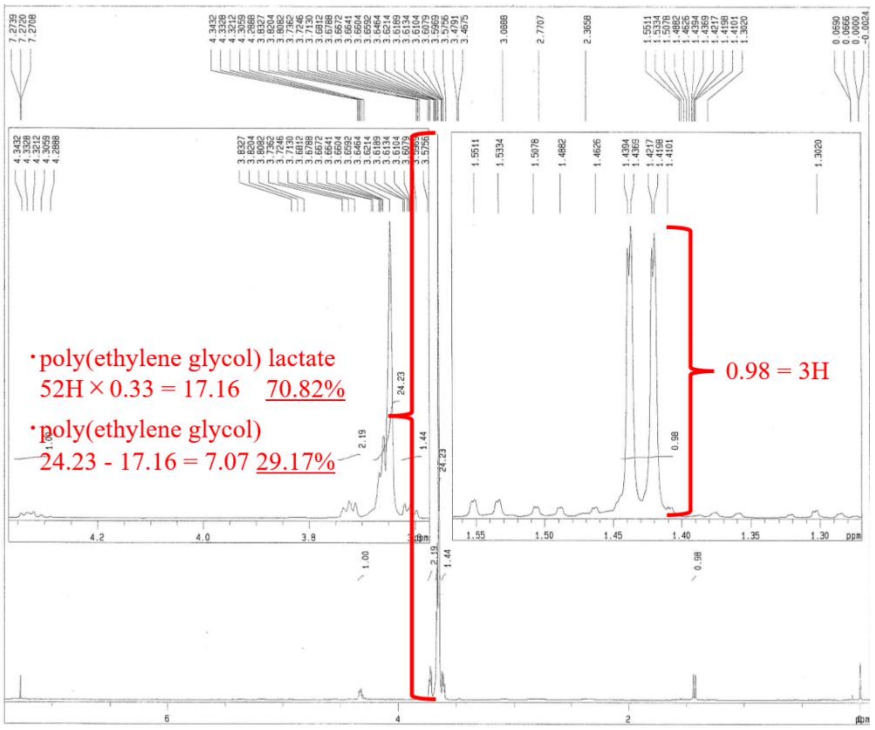

Figure S2. Calculation of the content of the products in the reaction mixture based on the integral intensity.

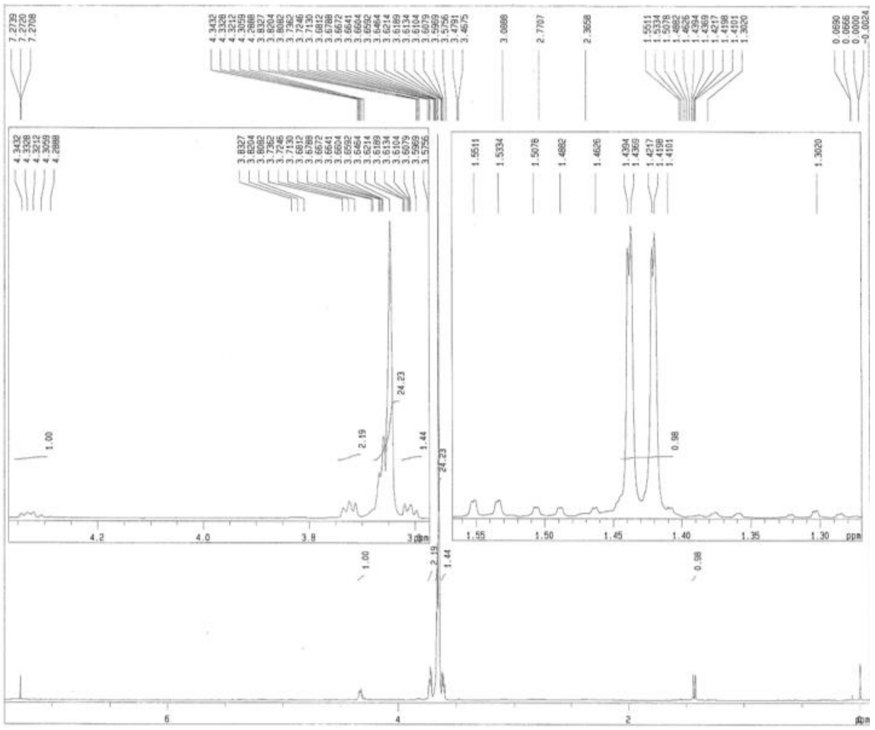

Figure S3. ^{1}H NMR spectrum of the reaction product obtained from poly(ethylene glycol) 600 and ethyl lactate.

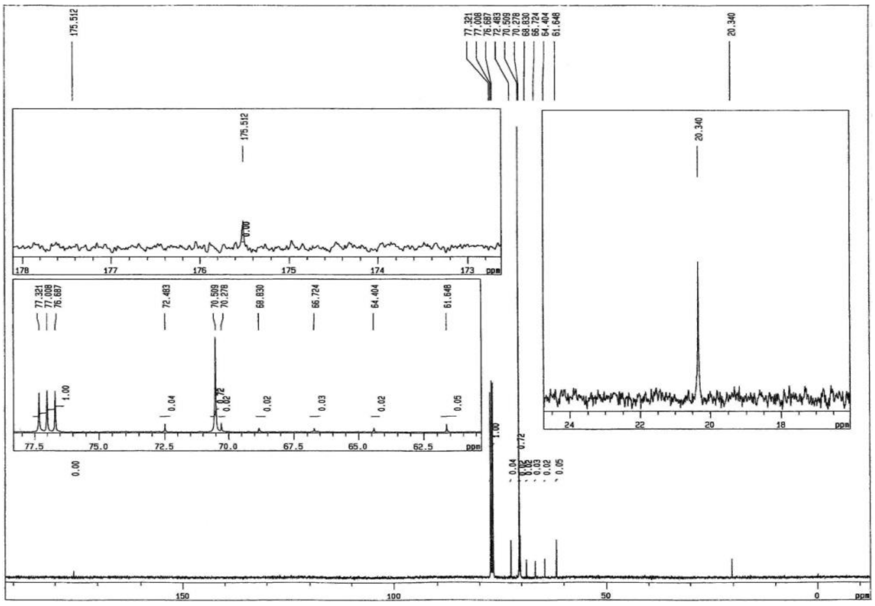

Figure S4. $^{13}C\{H\}$ NMR spectrum of the reaction product obtained from poly(ethylene glycol) 600 and ethyl lactate.

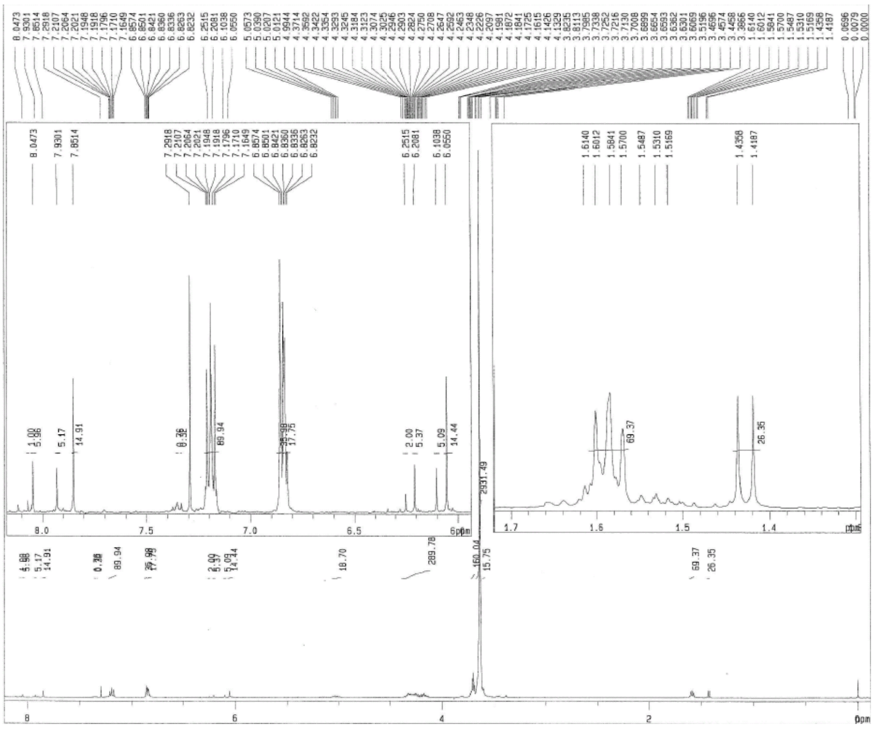

Figure S5. ^{1}H NMR spectrum of poly[(oxyethylene H-phosphonate)-*co*-poly(ethylene glycol)lactate H-phosphonate].

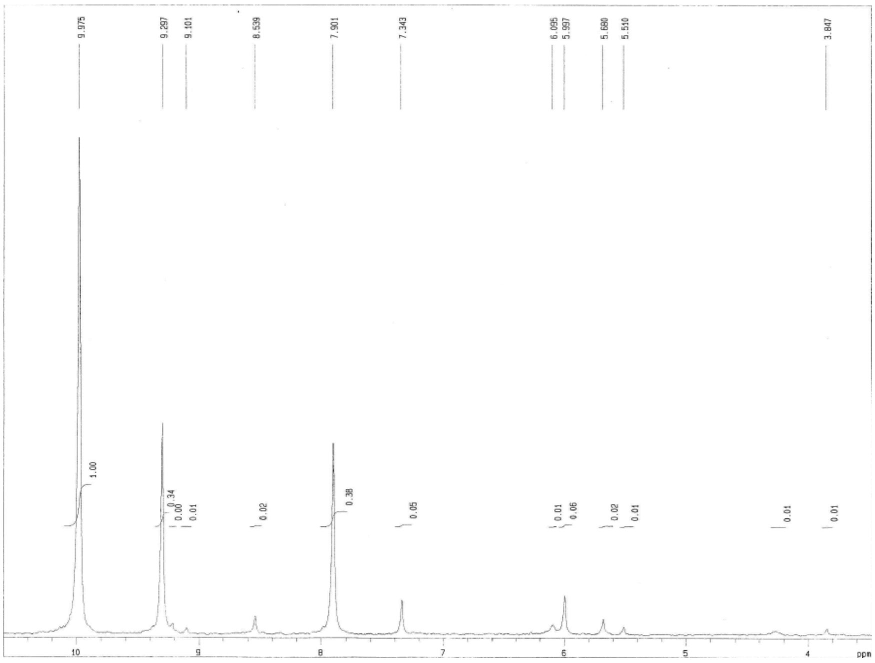

Figure S6. $^{31}P\{H\}$ NMR spectrum of of poly[poly(ethylene glycol H-phosphonate] *co* poly[poly(ethylene glycol)lactate H-phosphonate].

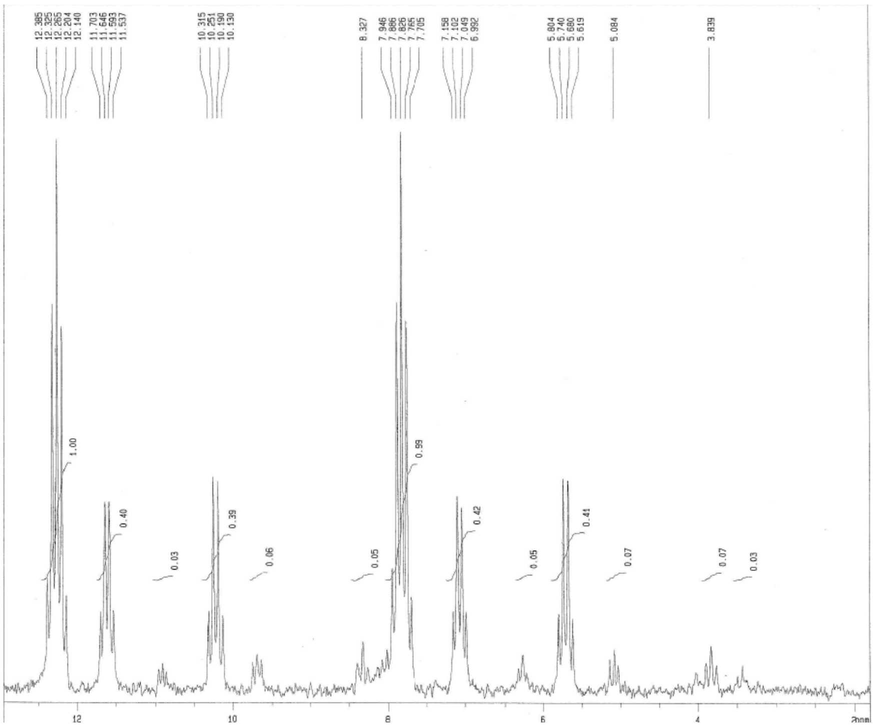

Figure S7. ^{31}P NMR spectrum of ofpoly[poly(ethylene glycol H-phosphonate] *co* poly[poly(ethylene glycol)lactate H-phosphonate].

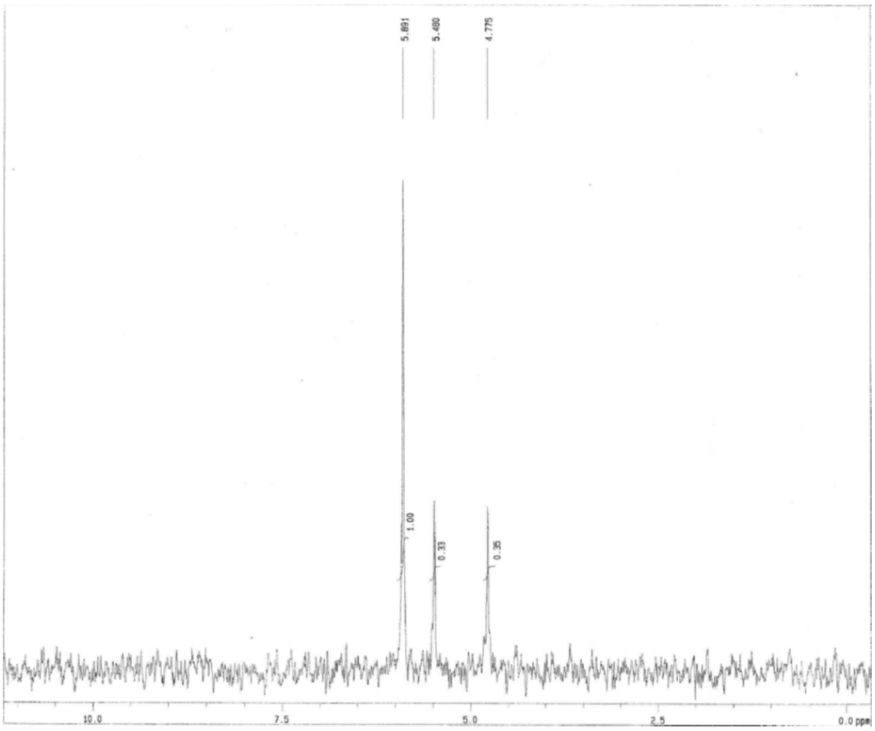

Figure S8. ^{31}P{H} NMR of poly[poly(ethylene glycol) chlorophosphate]*co* poly[poly(ethyleneglycol)lactate chlorophosphate].

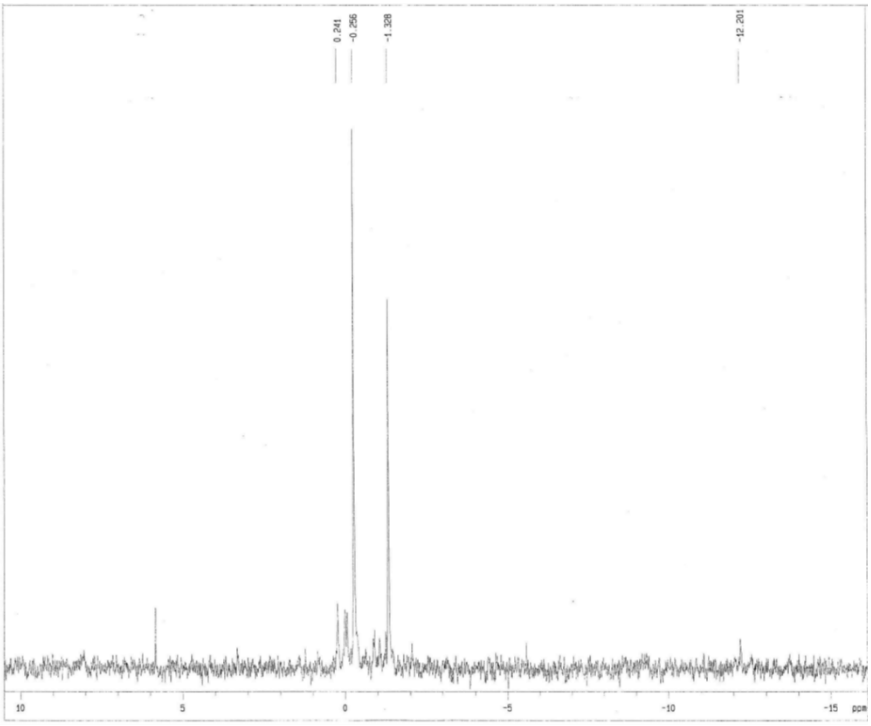

Figure S9. ^{31}P{H} NMR of poly[polyalklyl(ethylene glycol) phosphate]*co* poly[polyalkyl(ethyleneglycol)lactate phosphate].
